# Reliability and Validity of the Geriatric Depression Scale in Italian Subjects with Parkinson's Disease

**DOI:** 10.1155/2018/7347859

**Published:** 2018-08-01

**Authors:** Perla Massai, Francesca Colalelli, Julita Sansoni, Donatella Valente, Marco Tofani, Giovanni Fabbrini, Andrea Fabbrini, Michela Scuccimarri, Giovanni Galeoto

**Affiliations:** ^1^Sapienza University of Rome, Rome, Italy; ^2^Department of Public Health and Infection Disease, Sapienza University of Rome, Rome, Italy; ^3^Department Human Neurosciences, Sapienza University of Rome, Rome, Italy; ^4^IRCSS Neuromed Institute, Pozzilli, IS, Italy

## Abstract

**Introduction:**

The Geriatric Depression Scale (GDS) is commonly used to assess depressive symptoms, but its psychometric properties have never been examined in Italian people with Parkinson's disease (PD). The aim of this study was to study the reliability and validity of the Italian version of the GDS in a sample of PD patients.

**Methods:**

The GDS was administered to 74 patients with PD in order to study its internal consistency, test-retest reliability, construct, and discriminant validity.

**Results:**

The internal consistency of GDS was excellent (*α* = 0.903), as well as the test-retest reliability (ICC = 0.941 [95% CI: 0.886–0.970]). GDS showed a strong correlation with instruments related to the depression (*ρ* = 0.880) in PD (*ρ* = 0.712) and a weak correlation with generic measurement instruments (−0.320 < *ρ* <−0.217). An area under the curve of 0.892 (95% CI 0.809–0.975) indicated a moderate capability to discriminate depressed patients to nondepressed patient, with a cutoff value between 15 and 16 points that predicts depression (sensitivity = 87%; specificity = 82%).

**Conclusion:**

The GDS is a reliable and valid tool in a sample of Italian PD subjects; this scale can be used in clinical and research contexts.

## 1. Introduction

Parkinson disease (PD) is characterized by motor and nonmotor symptoms. Bradykinesia, tremor at rest, and rigidity are the cardinal motor manifestations of PD [1]. Nonmotor symptoms include gastrointestinal dysfunctions, sleep disorders, cognitive disorders, and neuropsychiatric disturbances. Depression has been found to be more frequent in PD patients than in age-matched healthy controls or in patients with other chronic medical conditions [2, 3]. For example, major depression may be found in up to 20% of PD patients [4]. To measure the level of depression, it is crucial that clinicians and researchers have access to reliable and valid instruments. A recent systematic review about depression tools in PD patients recommended the use of the Hamilton Depression Inventory as a rating scale, which takes into consideration the judgment of the clinician or the caregiver, and the Geriatric Depression Scale (GDS), that considers the patient's point of view, for the screening and measurement of the degree of perceived depression in patients with PD [5].

The GDS [6], composed by 30 items, was developed to evaluate the level of depressive symptoms over the past week. It was transculturally adapted in several languages [7–9], and it has proven to be reliable and valid in subjects with dementia [10–13], stroke [14–17], rheumatoid arthritis [18], and psychiatric disorders [19, 20]. In PD, several studies showed that GDS has good psychometric properties, a high internal consistency (Cronbach's alpha = 0.92) [21], an excellent test-retest reliability (intraclass correlation coefficient = 0.89 [95% CI 0.83–0.93]), and a minimal detectable change of 5.4 points [22]. Taking into account the validity, the GDS showed good correlations with the Beck Depression Inventory (*r*_s_=0.62, *p* < 0.05) and with mood related items of the Unified Parkinson's Disease Rating Scale (*r*_s_=0.38, *p* < 0.05) [23], and moderate correlations with the 17-item Hamilton Depression Rating Scale (*r*=0.54, *p* < 0.001) [24]. Recently, the GDS was used in an Italian sample of geriatric patients, and this study confirmed the good psychometric properties of GDS [25]. As the measurement properties of an instrument are affected by the disease investigated and by the contextual factors, for a reliable and valid use of the instrument in Italian subjects, the GDS should be validated also in the target population to which the questionnaire will be administered. No study has assessed the psychometric properties of GDS in Italian patients with PD. Therefore, the aim of this study is to assess the reliability and the validity of the GDS in a sample of Italian PD patients, using the Classical Theory Test.

## 2. Methods

### 2.1. Subjects

Seventy-four (older than 18 years) patients with clinically diagnosed PD were consecutively recruited through a convenience sample in the Rehabilitation Unit of San Giovanni Battista Hospital, Polyclinic Italia, and in the Department of Neurosciences, Sapienza University of Rome. Patients with cognitive impairment (Mini-Mental State Examination score <23 points) and problems with reading and understanding the Italian language were excluded. All subjects gave their informed consent [26, 27] to participate in the study, and the research was conducted according to the principles of Declaration of Helsinki.

### 2.2. Outcome Measures

#### 2.2.1. Geriatric Depression Scale

This scale assesses the depressive symptoms [6]. The version used in this study was composed by 30 items that investigated different aspects of the depression over the last week. Each item is rated by a dichotomous score (yes = 1; no = 0), and some items (Item numbers 1, 5, 7, 9, 15, 19, 21, 27, 29, and 30) presented a reverse score (yes = 0; no = 1). The total score is given adding the item scores, and it ranged from 0 (no depression) to 30 (maximum depression) points. The Italian version used in this study demonstrated to be reliable and valid [25].

#### 2.2.2. Hospital Anxiety and Depression Scale

This scale measures the level of depression and anxiety [28]. It is composed by 14 items divided in two subscales: 7 items investigate depressive symptoms, and the other 7 measure anxious symptoms. Subjects respond to each item on four-level ordinal score (0 = no symptoms; 3 = maximum symptoms); therefore, the total scores may vary between 0 and 21 points for each subscale. The Italian version of the scale was used in this study [29].

#### 2.2.3. Parkinson Disease Questionnaire

This questionnaire assesses the impact of parkinsonian symptoms in the life of these patients in the past month [30]. It contains 39 items that examine 8 domains through separately scored subscales: mobility (10 items), activities of daily living (6 items), emotional well-being (6 items), stigma (4 items), social support (3 items), cognition (4 items), communication (4 items), and bodily discomfort (3 items). A 5-point level score is attributed to each item (0 = never; 1 = occasionally/rarely; 2 = sometimes; 3 = often; 4 = always). A total score ranging from 0 (indicating best health status) to 100 (indicating worst health status) was calculated by summing the score of each item, both for the 8 subscores and for the total score. The Italian version used in this study was recently evaluated [31] and revealed good psychometric properties.

#### 2.2.4. Short Form 36-Health Survey Questionnaire (SF-36)

This is a 36-item questionnaire measuring the patient's health status in the past four weeks [32]. The total score ranges from 0 to 100 with higher scores indicating a better condition. The Italian version is considered to be a valid and reliable tool [33].

#### 2.2.5. Barthel Index

This well-known test measures the disability on the ADLs [34]. It is composed of 10 items including feeding, bathing, grooming, dressing, bowel and bladder control, toilet use, transfers (bed to chair and back), mobility, and stairs climbing. Three ordinal level scores are attributed to each item (0, 5, or 10; 15 points for items regarding transfers and mobility) to assess whether the patient can perform the various activities independently, with assistance or whether they are totally dependent from others. The total score is generated summing each score, and it varies from 0 (total dependence) to 100 (total independence). The Italian version was administered in this study [35, 36].

### 2.3. Procedures

Four clinicians (three occupational therapists and one physical therapist) screened all patients for their recruitment. Once enrolled, these clinicians collected demographic and clinical variables and administered the outcome measure to all patients. In order to study the test-retest reliability, the GDS was readministered after seven days. To assess the discriminant validity, a physician diagnosed the depression in this sample. According to DSM-5, patients were diagnosed with depression if they had at least five depressive symptoms including “depressed mood” and “loss of interest or pleasure” for at least two weeks [37].

### 2.4. Statistical Analysis

Descriptive statistics was used to analyze the sample characteristics; in particular, mean ± standard deviation (SD), median with 25th and 75th percentiles, and frequency with percentage were calculated for intervallic, ordinal, and categorical data, respectively.

The reliability of GDS was assessed in terms of internal consistency and test-retest reliability. Internal consistency was determined calculating Cronbach's alpha [38]: for values closer to 1, the internal consistency is higher. Alpha was considered excellent if >0.9, good if >0.8, and acceptable if >0.7 [39]. Test-retest reliability was calculated by the intraclass correlation coefficient (ICC) with a 95% confident interval (CI). ICC values greater than 0.75 are a minimum requirement to use the instrument in group measurements [40]; ICC values greater than 0.90 are considered essential for the use of the instrument in individual measurements [41].

The construct validity of the GDS was studied calculating the Pearson correlation coefficient (*ρ*) when comparing the GDS with the other administered instruments. The following ranges were considered in order to interpret the results: *ρ* > 0.70 = strong correlation, 0.50 < *ρ* < 0.70 = moderate correlation, and *e ρ* < 0.50 = weak correlation [42].

In order to study the discriminant validity, the receiving operating characteristic (ROC) curve was created, and the area under the curve (AUC) was calculated. The closer the AUC value is to 1.0, the greater the instrument's ability to distinguish depressed and nondepressed patients. An AUC higher than 0.75 confers to the tool a moderate discriminative validity; while an excellent one is demonstrated by a value ≥0.90.

For all statistical analyses, the *α* value was set at 0.05, and SPSS statistical software program, version 18.0 for Windows (SPSS Inc., Chicago, IL, USA), was used.

## 3. Results

### 3.1. Sample Characteristics

Seventy-four patients (44 males; 30 females) with PD were included in this study. The demographic and clinical characteristics of the patients studied are reported in Table 1.

### 3.2. Internal Consistency

The internal consistency for the total GDS score was excellent (*α* = 0.903).

### 3.3. Test-Retest Reliability

Test-retest reliability was assessed in a subsample of 35 patients. Excellent reliability was observed for the GDS total score (ICC = 0.941 [95% CI: 0.886–0.970]).

### 3.4. Validity

Pearson's correlation coefficient values are reported in Table 2. Taking into account the comparisons between GDS and the other instrument related to depression (HADS) and PD (PDQ-39), Pearson coefficient ranged between 0.712 and 0.880, indicating a strong correlation. On the other hand, regarding the comparisons between GDS and generic measurement instrument (Barthel Index and SF-36), the correlation coefficient varied from −0.320 to −0.217, showing a weak correlation.

**Figure 1:**
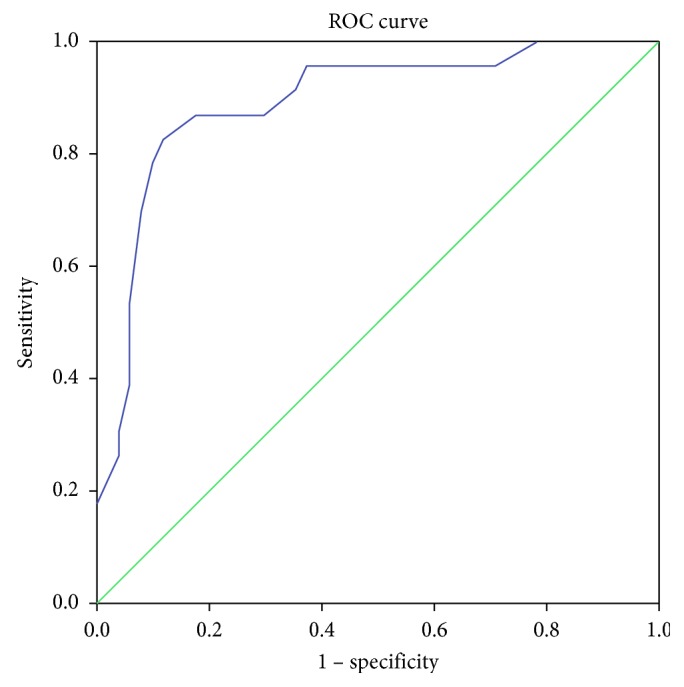
Receiving operating characteristic curve.

Regarding the discriminant validity, the AUC showed a value of 0.892 (95% CI 0.809–0.975), indicating a moderate capability to discriminate depressed patients to nondepressed patient. The score with the best sensibility and specificity that predicts depression is between 15 and 16 (sensitivity = 87%; specificity = 82%) (Figure 1).

## 4. Discussion

The use of a reliable and valid instrument is essential in clinical practice and when measuring specific outcomes [43]. Several questionnaires are available to measure depression in patients with PD [5]. The psychometric properties of GDS have been extensively studied in different pathologies and in different settings. To our knowledge, however, no study assessed the psychometric properties of GDS in Italian patients with PD. Studying the measurement properties in the context in which the instrument will be administered is crucial because these properties can be influenced by various contextual, social, and environmental factors [44]. The results of our study show that GDS is a reliable and valid instrument in Italian patients with PD.

The internal consistency assessed by calculating Cronbach's alpha (equal to 0.903) was excellent. The results obtained in the PD patients we studied are similar to those obtained in patients with different clinical conditions. For example, Cronbach's alpha was found to be 0.876 in a study on 294 geriatric patients [45] and 0.90 in 888 depressed and nondepressed elderly subjects [46].

We demonstrated an excellent test-retest reliability of the questionnaire (ICC = 0.941). The results obtained in our sample of PD patients are similar to those found in a cohort of 75 Chinese subjects with PD (ICC = 0.89 [95% CI 0.83–0.93]) [22].

The construct validity was investigated through the correlations between the GDS and other validated questionnaires. In particular, a strong construct validity was obtained through correlations with HADS (both with anxiety and depression) and PDQ-39. On the other hand, a weak correlation was found when the GDS was compared with the Barthel Index and the SF-36. The strong correlations between GDS and HADS can be explained because these two scales intend to measure the same variable, that is, the depression; these results are in line with previous studies that obtained similar correlations with questionnaires related to depression—Beck Depression Inventory (*r*_s_=0.62, *p* < 0.05) [23] and Hamilton Depression Rating Scale at 17 items (*r*=0.54, *p* < 0.001) [24]. Conversely, the low correlation found with SF-36 and Barthel Index may be explained because both the Barthel Index and the SF-36 are generic instruments.

Finally, the discriminating validity was studied through the ROC curve in order to identify the best sensitivity and specificity of the cutoff value that can distinguish depressed and nondepressed patients. The cutoff value of 15–16 points showed a sensitivity of 87% and a specificity of 82%. Comparing our results with those obtained in other studies is not easy considering the different patient populations and the different settings; for example, the study by McDonald et al. showed a cutoff value of 9–10 points [24] and the study by Ertan et al. [7] a cutoff value of 13–14.

This study presents limitations that need to be taken into account. The design of the study did not allow the assessment of some fundamental psychometric properties such as content validity and responsiveness.

In conclusion, this study shows that GDS can be used in clinical practice as a valid measurement instrument in order to quantify depression in patients with PD.

## Figures and Tables

**Table 1:** Main demographic and clinical characteristics of the sample (*N*=74).

Variables	Values
*Age (years)* ^a^	66.9 ± 9.7
*Gender* ^b^	
(i) Male	44 (59.5%)
(ii) Female	30 (40.5%)
*Depression* ^b^	
(i) Presence	23 (31.1%)
(ii) Absence	51 (68.9%)
*Medications prescribed to depressed subjects (N=23)* ^b^	
(i) Antidepressant	11 (47.8%)
(ii) Anxiolytic	10 (43.5%)
(iii) No medications	2 (8.7%)
*Educational level* ^b^	
(i) Primary	9 (12.2%)
(ii) Secondary	17 (23%)
(iii) High school	33 (44.6%)
(iv) Degree	13 (17.6%)
(v) Not reported	3 (4.1%)
*Employment* ^b^	
(i) Employed	13 (17.6%)
(ii) Not employed	4 (5.4%)
(iii) Retired	57 (77%)
*Marital status* ^b^	
(i) Married	56 (75.6%)
(ii) Unmarried	17 (23%)
(iii) Not reported	1 (1.4%)
Time since PD diagnosis (years)^a^	7.8 ± 5.6
Hoehn and Yahr stage^c^	3 (2; 3)
*Setting* ^b^	
(i) Department	20 (27%)
(ii) Ambulatory	53 (71.6%)
(iii) Day-hospital	1 (1.4%)
MMSE score^c^	29 (27.25; 30)
HADS-A score^c^	7 (4; 10)
HADS-D score^c^	7 (4; 10)
HADS total score^c^	15 (10; 20)
GDS total score^c^	13 (6; 19)
*PDQ-39 subscale score* ^c^	
(i) Mobility	17.5 (7.5; 25.75)
(ii) Activities of daily living	10 (4; 15.75)
(iii) Emotional well-being	9 (5; 14)
(iv) Stigma	4 (2; 8)
(v) Social support	1 (0; 3.75)
(vi) Cognition	5 (2; 8)
(vii) Communication	3 (1.25; 6)
(viii) Bodily discomfort	4 (2; 7)
*PDQ-39 total score* ^c^	59 (31.25; 76)
SF-36^c^	95 (86.25; 102)
Barthel Index^c^	85 (75; 95)

Data are expressed as ^a^mean ± standard deviation, ^b^frequency with percentage, or ^c^median with 25th and 75th percentiles. MMSE: Mini-Mental State Examination; HADS-A: Hospital Anxiety and Depression Scale of Anxiety; HADS-D: Hospital Anxiety and Depression Scale of Depression; GDS: Geriatric Depression Scale; PDQ-39: Parkinson's Disease Questionnaire; SF-36: Short Form 36-Health Survey Questionnaire.

**Table 2:** Pearson's correlation coefficient for each comparison.

	HADS-A	HADS-D	Total HADS	PDQ	SF-36	Barthel Index
GDS	0.799^*∗*^	0.800^*∗*^	0.880^*∗*^	0.712^*∗*^	−0.320^*∗∗*^	−0.217

^*∗*^ *p* < 0.01; ^*∗∗*^*p* ≤ 0.5. HADS-A: Hospital Anxiety and Depression Scale of Anxiety; HADS-D: Hospital Anxiety and Depression Scale of Depression; GDS: Geriatric Depression Scale; PDQ-39: Parkinson's Disease Questionnaire; SF-36: Short Form 36-Health Survey Questionnaire.

## Data Availability

The data used to support the findings of this study are available from the corresponding author upon request.
